# Six personas to adopt when framing theoretical research questions in biology

**DOI:** 10.1098/rspb.2024.0803

**Published:** 2024-09-18

**Authors:** Allison K. Shaw, Ave T. Bisesi, Chris Wojan, Dongmin Kim, Martha Torstenson, Naven Narayanan, Peter Lutz, Ruby Ales, Cynthia Shao

**Affiliations:** ^1^ Department of Ecology, Evolution and Behavior, University of Minnesota, St Paul, MN 55108, USA; ^2^ Department of Computer Science, University of Minnesota, Minneapolis, MN 55455, USA; ^3^ Department of Mathematics, University of Minnesota, Minneapolis, MN 55455, USA; ^4^ Department of Biochemistry, University of Minnesota, Minneapolis, MN 55455, USA

**Keywords:** mathematical biology, methodology, narratives, pedagogy, scientific writing, theoretical ecology

## Abstract

Theory is a critical component of the biological research process, and complements observational and experimental approaches. However, most biologists receive little training on how to frame a theoretical question and, thus, how to evaluate when theory has successfully answered the research question. Here, we develop a guide with six verbal framings for theoretical models in biology. These correspond to different personas one might adopt as a theorist: ‘Advocate’, ‘Explainer’, ‘Instigator’, ‘Mediator’, ‘Semantician' and ‘Tinkerer’. These personas are drawn from combinations of two starting points (pattern or mechanism) and three foci (novelty, robustness or conflict). We illustrate each of these framings with examples of specific theoretical questions, by drawing on recent theoretical papers in the fields of ecology and evolutionary biology. We show how the same research topic can be approached from slightly different perspectives, using different framings. We show how clarifying a model’s framing can debunk common misconceptions of theory: that simplifying assumptions are bad, more detail is always better, models show anything you want and modelling requires substantial maths knowledge. Finally, we provide a roadmap that researchers new to theoretical research can use to identify a framing to serve as a blueprint for their own theoretical research projects.

## 1. Introduction

Theory is a critical component of how biology (and science broadly) is conducted, and complements experimental and observational approaches. Theory serves many purposes, including to explore the logical consistency of ideas, to identify the simplest model that can predict observed phenomena, to demonstrate the complexity of a situation, to suggest ways of looking at empirical data, to generate novel hypotheses and to explore possible ranges of behaviour of a system [1,2]. Theory can take a range of forms including verbal, conceptual, computational and mathematical. Broadly, theory serves as scaffolding [3] that helps us make sense of observations and experiments. Yet, papers with primarily theoretical approaches make up a small portion of the overall biological literature; only 18% of papers in the most theory-heavy journals within ecology and evolutionary biology presented primarily theoretical findings [4,5]. Perhaps due to this small representation, most biologists receive little training on how to design and interpret theory (particularly mathematical theory) [6], especially compared with the amount of training they receive on experimental approaches.

Lack of training could result from an absence of conversation among biologists about best practices for designing and interpreting theory. However, this is clearly not the case; for example, people have debated how to do theory for as long as theoretical ecology has been a field. Levins’ seminal paper [7] argued that the three key aims for models are realism, precision and generality. Since no model can accomplish all three aims simultaneously, we need different sets of models to prioritize different aims so that we can find true understanding at the point(s) where the results intersect [7]. May [8] cautioned against having an uneven balance of detail in models; including extensive detail in some model aspects while keeping others vague can convey a false sense of how much realism the model includes. In contrast to Levins and May, some researchers have called for prioritizing more of one type of theoretical model over others. For example, Holling [9] argued that the field had enough of what he called ‘strategic’ models (that sacrifice precision to focus on generality), but needed more ‘tactical’ models. Evans *et al*. [10] similarly called for embracing complex models as a means of achieving generality through generating testable predictions. The opposite argument has also been made: Marquet *et al*. [11] called for the development of more ‘efficient’ theories that have fewer parameters and do not need to be precise. In addition, much has been written arguing for the value of theory in biology as a whole, drawing parallels between how theoretical and empirical studies are conducted in both ecology [1] and evolutionary biology [12]. Yet, conversations within these fields about designing and interpreting theory have not translated into guidance for newcomers on how to conduct and communicate theory. This lack of guidance creates a barrier for scientists new to theoretical research.

In response, there have been a number of recent ‘how to’ guides aimed at breaking down this barrier for researchers working with theoretical approaches. For example, recent guides on how to communicate theory to broad audiences include suggestions like clearly stating context and assumptions, reducing irrelevant complexities (adjusting maths to the target audience), using clear and standardized mathematical notation, and using analogies and narratives to facilitate links between new and existing information [13,14]. In another how to guide, Edwards and Auger-Méthé [15] provide advice for choosing mathematical notation. There have also been recent guides on how to read and use mathematical theory in ecology. Shoemaker *et al*. [13] suggest that readers spend extra time engaging with the maths, including breaking down equations into components and working through them with peers, connecting specific equations to a general class of models and reconstructing models or exploring parameter space to get a better handle on them. Other guides show how to use theoretical frameworks to guide empirical laboratory and field work, use mathematical equations to make empirically based calculations and test either the assumptions or predictions of theory [16,17]. Overall, these suggestions provide guidance for researchers who are either reading broadly before they start a project, or have completed a theoretical research project and want to communicate it clearly. In contrast, there is less guidance for the middle stage in the theory development process: how to choose—and frame—a theoretical research question. This is a critical gap. Even researchers who never pose theoretical research questions themselves will use and evaluate theory (e.g. as guides for experimental or observational work). Thus, we all benefit from understanding how theory is framed in order to help evaluate when theory has been successful.

Here, we fill this gap by providing guidance for how to frame theoretical research in biology. Theoretical research often starts out as a verbal model, using reasoning to set up an argument about what is expected to occur. Verbal arguments can only get us so far, and relying on common sense and intuition often leads us astray [18]. It is at these points that turning the verbal (or narrative-based) argument into a mathematical (or computational) form can provide clarity and help extend a verbal argument [19]. Here, we argue that the converse is also true: a clear verbal framing can help improve the usefulness of a mathematical or computational model. Below, we present six ways to frame theoretical research, describing each as a persona one might adopt as a theorist: the ‘Advocate’, ‘Explainer’, ‘Instigator’, ‘Mediator’, ‘Semantician’ and ‘Tinkerer’. We show that these personas are not mutually exclusive; the same question or idea can be framed using multiple personas, and the appropriate choice will often depend on the research goal or intended audience. Finally, we demonstrate how the ways of thinking that we present can be used to address common misconceptions about theory.

## 2. Framing theory

**Figure 1.**
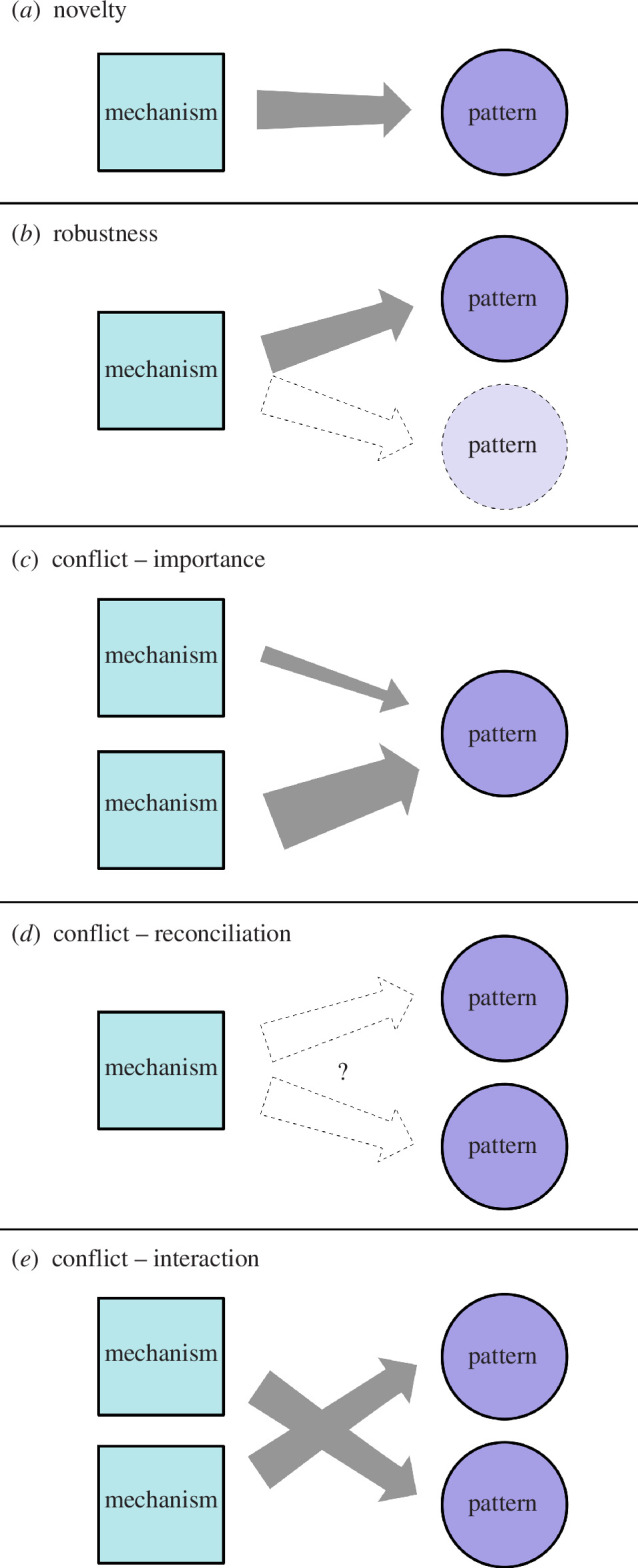
There are many possible links between pattern(s) and mechanism(s), depending on whether one or more pattern(s) and mechanism(s) are considered: (*a*) a novel link between one mechanism and one pattern, (*b*) the robustness of one mechanism leading to multiple patterns, (*c*) the relative importance of multiple mechanisms for one pattern, (*d*) the reconciliation of multiple patterns with one mechanism and (*e*) the interaction among multiple mechanisms to generate multiple patterns.

Theoretical models are fundamentally about understanding the link between outcomes and assumptions [20]. Those assumptions will include what biological details to focus on and what to ignore. In particular, many models aim to link ‘patterns’ (outcomes of interest) to ‘mechanisms’ (processes that can generate those outcomes) (figure 1). When a researcher starts developing a theoretical model, they typically have a sense of both what mechanism(s) they want to include and what pattern(s) to expect. The core part of the modelling process is concretely stating the specifics of patterns and mechanisms and determining the conditions under which mechanisms and patterns are linked. Here, we propose that when writing about theory for general readers (e.g. in grant proposals or manuscripts), it can help to focus on either the pattern or mechanism as a starting point and then connect it to the other. For example, a theoretical project could start by describing a pattern and develop theory to better understand the mechanisms that cause it (i.e. exploring causes). Alternatively, a theoretical project could start by considering a mechanism and use theory to better understand the patterns it can generate (i.e. exploring consequences). In addition to these two starting points, we suggest that the goal of theory can be pitched with a specific focus: novelty, robustness or conflict. Theory with a novelty goal presents new (or previously overlooked) ideas, while robustness-oriented theory aims to examine the boundaries and context dependence of previously described relationships, and theory focused on conflict endeavours to evaluate contradictory observations or opposing explanations. Taken together, these two starting points and three foci lead to six different ways to frame theory, or six different personas one might adopt as a theorist (table 1).

**Table 1.** Overview of six different ways to frame theoretical models: either start with a pattern or mechanism (rows) and focus on novelty, robustness or conflict (columns). For each framing, there is a corresponding persona one can adopt as a theorist.

	novelty	robustness	conflict
**start with pattern**	the ‘Explainer’: here’s a pattern, can—and under what conditions does—this mechanism generate it?	the ‘Tinkerer’: here’s a pattern, how robust is it to changes in the underlying mechanism(s)?	the ‘Mediator’: here are multiple conflicting empirical data patterns, how do we reconcile them? here are multiple conflicting conceptual ideas, how do we reconcile them? here is a pattern in data that doesn’t match the existing theory, how do we reconcile this?
**start with mechanism**	the ‘Advocate’: here is a mechanism that has been overlooked, what are the consequences of including it? here is a mechanism at one scale, when does it affect patterns at another scale?	the ‘Semantician’: here is a mechanism, how does its effect on the pattern depend on how the mechanism is captured in a model?	the ‘Instigator’: here are multiple mechanisms, what is their relative importance for generating a pattern? here are multiple mechanisms, how might they interact to generate different patterns?

**Figure 2.**
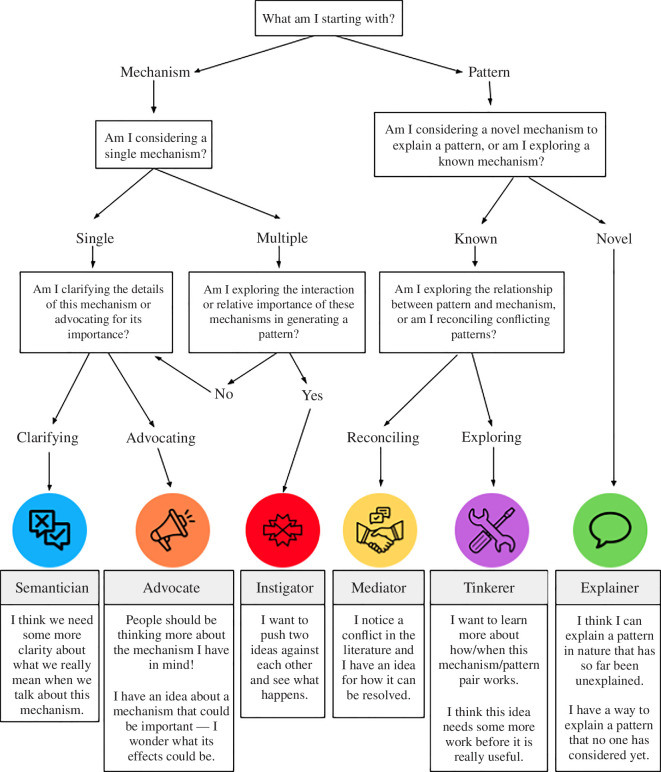
Roadmap to help researchers choose a framing for their research question. Explore this roadmap as a quiz at https://z.umn.edu/theorypersona.

These six personas form the core of our paper. We present them in several different ways (i.e. text, tables, figures) below, to maximize the accessibility of our categorization scheme. We also imagine that the same person may find different ways of accessing information useful at different points within their project, thus revisiting different sections. In the remaining text of §2, we explain the six different personas in detail. To illustrate each persona, we give examples of specific research questions, drawing primarily on recently published theory in ecology and evolutionary biology from three places: (i) searching for theory papers from the year 2022 in journals from our field (*Ecology Letters*, *Evolution*, *Journal of Animal Ecology*, *Oikos*, *Proceedings of the Royal Society B*, *The American Naturalist*, *Theoretical Ecology*), (ii) looking at the set of theory papers analysed in Servedio [21] and (iii) previous papers by ourselves (the authors). We expect this section may be most useful for those new to the ideas presented here. In table 1, we organize the six personas by their starting point (pattern, mechanism) and focus (novelty, robustness, conflict) and provide general ways to frame research questions for each. This section may be useful for researchers who want to brainstorm multiple different theoretical questions for their research project. In figure 1, we diagram the different relationships between mechanism(s) and pattern(s), which depend on the focus (novelty, robustness, conflict) and on whether there is one or more of each pattern and mechanism. In figure 2, we provide a decision tree that a researcher can work through by answering a series of questions, which lead to one of the six personas with examples of attitudes one might have as each of these personas (also available as a quiz at https://z.umn.edu/theorypersona). This figure may be most useful for researchers looking to narrow in on one particular way to frame their question.

### (a) Starting with pattern, focusing on novelty (the ‘Explainer’)

Theory can be motivated as providing a novel mechanism to *explain* a particular pattern. In this persona, one could frame a question like, ‘Here is a pattern, can—and under what conditions does—this mechanism generate it?’ For example, the pattern of species coexistence is often viewed from a lens of competitive interactions between species [22]. A recent paper by McPeek *et al*. [23] explored how the mechanism of mutualistic relationships could foster coexistence between competitors by asking, ‘Under what conditions can mutualistic relationships between two competitors lead to their coexistence?’ As a second example, consider the observed pattern that some spreading populations fluctuate in how fast they spread over time, expanding quickly in some years and expanding slowly (or even contracting) in others. This pattern is often attributed to environmental heterogeneity or stochasticity [24,25]. Sullivan *et al*. [26] developed a model to show how the mechanism of deterministic internal population dynamics alone could generate fluctuations in spreading speed, by asking, ‘Under what conditions can fluctuations in spreading speed occur in deterministic models with spatially and temporally constant environments?’.

### (b) Starting with pattern, focusing on robustness (the ‘Tinkerer’)

Alternatively, theory can be motivated by testing the robustness of a particular pattern. In this case, the role of the theorist is to explore the effects of *tinkering* with existing models. Questions with this framing could be, ‘Here’s a pattern, how robust is it to changes in the underlying mechanism(s)?’ This persona is typically associated with clarifying a mechanism (rather than proposing a new one), either by making a verbal model more concrete, or by clarifying the conditions under which a mechanism can generate a pattern. For example, Shaw *et al*. [27] used one type of modelling approach (ordinary differential equations) to study the relative importance of insect life-history and insect behaviour on the spread rate of vector-borne plant pathogens (pattern). Following this, Shaw *et al*. [28] studied how robust this pattern was to the modelling approach used by developing an individual-based model to capture the same mechanisms of the same system.

### (c) Starting with pattern, focusing on conflict (the ‘Mediator’)

Third, theory can be motivated by needing to reconcile conflicting patterns—within the empirical literature, between conceptual ideas, or between theoretical and empirical results. The goal of this type of theory is to *mediate* a conflict in patterns. Unlike the above two (‘Explainer’, ‘Tinkerer’), this persona is motivated by several patterns rather than a single one. Questions here could be framed accordingly, e.g. as ‘Here are multiple conflicting empirical data patterns, how do we reconcile them?’ For example, some empirical studies find the pattern that migratory individuals typically have greater parasite infection than resident individuals [29,30], while other studies find the opposite pattern [31,32]. Shaw *et al*. [33] used a theoretical model to clarify the mechanism leading to these disparate patterns: migration can simultaneously lead to a higher richness of parasites and lower infection prevalence. Thus, studies that use richness as a metric find one pattern, while studies that use prevalence find the opposite.

Questions could also be framed as, ‘Here are multiple conflicting conceptual ideas, how do we reconcile them?’ For example, studies that aim to understand sex-specific patterns of how individuals move to find mates in sexually reproducing species have generated two contradicting predictions. Theory that focuses on mate finding shows that when individuals of one sex move more, individuals of the other sex should move less, creating sex bias in dispersal [34]. In contrast, theory that focuses on dispersal distance shows that when dispersal affects the potential to find mates, selection favours both males and females to have similar dispersal kernels, creating no sex bias in dispersal [35]. Shaw and Kokko [36] built a single model to resolve this contradiction, showing that the mechanism details shape which results apply—e.g. if females moving more brings females closer to males (and thus males do not have to move), we get one outcome, whereas if females moving more leads to males being left behind, we see the other outcome.

A final way that questions could be framed using this persona is as, ‘Here is a pattern in data that does not match the existing theory, how do we reconcile this?’ For example, an observed empirical pattern is that seabirds have a diversity of foraging strategies [37], which conflicts with theory suggesting that there should be a single optimal foraging strategy [38]. Jeffries *et al*. [38] reconciled this conflict by developing a model where seabirds have only partial knowledge of the patchy food distributions in their environment and showed that different foraging strategies are favoured by birds with different personalities along the bold-shy continuum.

### (d) Starting with mechanism, focusing on novelty (the ‘Advocate’)

In contrast to the three personas above (‘Explainer’, ‘Tinkerer’, ‘Mediator’), theory can be framed as starting with a mechanism and determining the consequences in terms of the pattern (or patterns) generated. This kind of theory might result in *advocating* for an overlooked mechanism to be included. Theory that aims to describe a novel consequence of a mechanism could be framed as, 'Here is a mechanism that has been overlooked, what are the consequences of including it?' For example, Rabajante [39] advocates for considering the variance (rather than just the mean) of parasite burdens when modelling disease spread, showing that failing to do so can lead to either over- or underestimating patterns of parasite transmission. As a second example, Yamamichi and Letten [40] showed how adding a new mechanism (rapid evolution) to a model of species competing in the face of temporal fluctuations led to a broader set of conditions where the species could coexist (pattern).

This persona can be particularly effective at connecting scales in ecology by asking, 'Here is a mechanism at one scale, when does it affect patterns at another scale?' For example, Shoemaker *et al*. [41] developed a model to show that the preferences that aphids have for feeding on different host plants (mechanism at an individual scale) can shape how fast plant pathogens (which are transmitted by aphids) spread across a host plant population (pattern at the population scale). They use their model to identify the contexts in which the feeding preference mechanism they advocate for is important.

### (e) Starting with mechanism, focusing on robustness (the ‘Semantician’)

Just like the ‘Tinkerer’ persona above, theory that starts with a mechanism can also have a focus on robustness by asking, 'Here is a mechanism, how does its effect on the pattern depend on how the mechanism is captured in a model?' This persona is fundamentally focused on the *semantics* of a mechanism, that is, how the mechanism is expressed in the model(s). For example, previous work has shown that species with environmental sex determination are particularly sensitive to environmental change [42,43]. Crowley and Labonne [44] developed a model to ask, 'How robust are these conclusions to how evolutionary success (i.e. fitness) is encoded?' In this model, the authors measured how environmental quality and habitat loss (multiple mechanisms) could influence the fitness of populations (pattern) based on whether the model was formulated in terms of growth rate (*r*) or lifetime reproductive success (*R*_0_). Thus, such models can decipher how robust outcomes (or patterns) are to underlying differences in descriptions of the same mechanisms in different models.

### (f) Starting with mechanism, focusing on conflict (the ‘Instigator’)

Finally, theoretical models can be used to explore the consequences of several mechanisms in combination. In this kind of framing, the theorist *instigates* a potential conflict (or interaction) between multiple mechanisms and investigates the outcome. The question here could be framed in terms of relative importance, e.g. ‘Here are multiple mechanisms, what is their relative importance for generating a pattern?’ For example, abundance of grazers can dramatically impact reef cover dynamics [45]. Dispersal of coral and macroalgae between reefs seems likely to be important for reef cover dynamics as well [46], yet it has been less explicitly explored. Greiner *et al*. [47] built a theoretical model to ask, 'What is the relative importance of these two mechanisms?'. Here, the authors study the relative effects of grazer and dispersal behaviour (multiple mechanisms) which interact to influence spatial patterns of coral reefs (pattern).

Alternatively, questions in this persona could be framed as combining several mechanisms that have previously been considered separately to ask, ‘Here are multiple mechanisms, how might they interact to generate different patterns?’ For example, studies have examined how seed dormancy [48] and facilitation [49] each separately shape plant populations and communities. Leverett and Shaw [50] developed a theoretical model with both mechanisms, in order to understand how facilitation between seedlings interacts with dormancy in seeds to shape patterns of plant population dynamics. Another instance of an ‘Instigator’-style model comes from Shoemaker and Melbourne [51], who developed a framework with multiple ecological mechanisms thought to influence the pattern of spatial coexistence of species in competitive metacommunities. Using their framework, they calculated the relative contribution of these different ecological mechanisms in determining the strength of coexistence (here, a species’ low-density growth rate) for different metacommunity paradigms.

### (g) Other perspectives

Although the six personas as described in detail above form the core of our paper, we wanted to comment on a few other aspects of framing theory that readers might find useful. First, one could start by asking how many mechanisms and how many patterns they were interested in, and use that to choose a persona (figure 1). A single mechanism and single pattern would best fit one of the novelty personas of ‘Advocate’ or ‘Explainer’. A single mechanism with two (or more) patterns would fit either robustness persona (‘Tinkerer’, ‘Semantician’) or the ‘Mediator’, while a single pattern with two (or more) mechanisms would fit the ‘Instigator’ persona of looking at the relative importance of the mechanisms. Multiple patterns and multiple mechanisms could also fit the ‘Instigator’ persona of aiming to understand the interaction among multiple mechanisms.

Second, the process of model development can be seen as the act of ‘translating’ mechanisms from the complex language of biology to the more abstract language of mathematics, analysing the model and then translating the results back again to understand the biological pattern produced [20]. Just like translation of text across different languages, there can be multiple ‘correct’ ways to translate between maths and biology. For example, a single biological system could be translated into, e.g. a differential equation model or into an individual-based model. Similarly, the same model result could be interpreted in the context of, e.g. metapopulations or in the context of disease dynamics. Viewing theory development as translation, we can see that some of the personas described above effectively compare multiple translations. For example, one could start with a biological pattern and see if the pattern is disrupted if the same biological mechanism is translated in two different ways in the model (the ‘Semantician’). Similarly, one could start with a biological mechanism and vary how it is translated into a model to see whether the details of translation affect the pattern generated (the ‘Tinkerer’). In contrast, the remaining personas (the ‘Advocate’, ‘Explainer’, ‘Instigator’, ‘Mediator’) are more often about developing a single translation from biology to maths and back again.

## 3. Flexibility in framing

In §2 above, we described each research project using a single persona. However, the same project can often be framed in several different ways. Indeed, many theoretical papers use several personas when describing their study (e.g. in the abstract versus the discussion). For other projects, the persona used at the start of the project may not be the same as the one used at the project’s end. Finally, the persona used might depend on the intended audience. For example, the persona used when writing a grant proposal might differ from the persona used when the work is published, and even the best persona to use might depend on the specific journal and its audience. In this section, we show how the same theoretical project can be framed using multiple personas.

Different ways of framing theoretical projects might be best suited to practitioners in different fields. For example, land and wildlife managers may be interested in starting with patterns to frame a theoretical project based on what they see in nature (e.g. observing the pattern that two plant species X and Y often coexist in a community that is affected by drought). In contrast, developing a theoretical project starting with a mechanism may be more relevant to ecologists who are interested in understanding the effects of potential factors that influence ecological systems (e.g. asking how the mechanism of drought affects the coexistence of plant species X and Y). The persona used will also depend on what models and insights already exist within a field of study. By providing examples of different ways to frame the same theoretical project, we hope to inspire practitioners to continue asking their research questions using different framings.

### (a) Multiple ways to start with pattern (Sullivan *et al*. 2017, *Proceedings of the National Academy of Sciences*)

First, consider a theoretical project that starts with a pattern of interest and aims to understand the mechanisms underlying it. In Sullivan *et al*. [26], the pattern of interest was species invasion dynamics and the large variation in speed of invasion (or range expansion) observed in many empirical systems. Understanding variation in speed of invasive species spread is crucial to devise strategies for their elimination or mitigation. In order to explain the pattern of varying invasive speeds for species, Sullivan *et al*. focused on two mechanisms: (i) a strong Allee effect (where populations cannot grow from low density) and (ii) fluctuations in population size due to overcompensatory growth or density dependence in dispersal. By building spatio-temporal mathematical models incorporating these mechanisms, the authors showed how demographic and dispersal dynamics jointly produced fluctuating invasion speeds as the population spread into new territory. Thus, framing the research question as an inquiry into mechanisms responsible for generating patterns of invasion speed variation led to novel scientific insights (i.e. pattern/novelty framing—the ‘Explainer’). However, one could approach the same project with a different framing: how robust is the pattern of variable invasion speeds found in nature to the specific forms of population dynamics and dispersal used in a model? Here, one could consider different existing population dynamics models (e.g. logistic growth, Allee effect) and study their interplay with different dispersal behaviours (e.g. dispersal propensity, dispersal distance). In this framing, the model could be viewed as the ‘Tinkerer’ persona (i.e. pattern/robustness). Finally, one could frame a question as the ‘Mediator’ persona (i.e. pattern/conflict) by noting that some invasive species spread at relatively constant rates. Here, the question could be: how do we reconcile the fact that the spread rate of some species fluctuates while the spread rate of others does not? While the methods and results section in both framings could remain identical, the motivation for the study would appear significantly different based on which above framing is used.

### (b) Multiple ways to start with mechanism (Miller *et al*. 2021, *Ecology Letters*)

Next, consider a theoretical project that starts with a mechanism of interest and aims to understand the pattern(s) it can generate. Miller *et al*. [52] used a theoretical model to understand the effects of disturbance history (a mechanism) on plant community structure and invasion outcomes (patterns), given two resident plant species and an invader species population (i.e. mechanism/novelty—the ‘Advocate’). However, the most relevant way to define disturbance history in a model may depend on what aspects of disturbance ecologists are interested in understanding. For example, one may be interested in disturbance intensity (i.e. the strength of the disturbance force) [53]. In contrast, one may be interested in disturbance frequency, which captures the number of disturbed events that have occurred per specified time-period [52]. Given different possible definitions of disturbance history, one could alternatively frame the project by asking how robust the model outcomes are to how the mechanism of disturbance history is defined in the model (i.e. mechanism/robustness framing—the ‘Semantician’). Alternatively, if disturbance history is viewed as the sum of several different elements, such as disturbance intensity and frequency, the same theory can be framed by understanding how two different mechanisms (disturbance intensity and frequency) combine to shape the dynamics of species community structure (i.e. mechanism/conflict framing—the ‘Instigator’). Thus, this same theoretical project could be framed in multiple ways that link these specific mechanisms to patterns in plant communities.

### (c) Changes in theoretical framing from start to finish

Finally, the framing used by researchers might change throughout the course of a research project. For example, Shaw and Binning [54] showed how migration from one environment to a second (migratory) environment could be used as a strategy to escape and recover from environmental-specific parasites in the first environment. When presenting this research in talks, a common audience question was 'What about parasites that are present in the migratory environment?' To answer this, the authors built a model that included parasites in each environment in order to understand how the costs/risks of having parasites present in both environments shaped the evolution of migration. Walking through our roadmap (figure 2), this would be a case of starting with a mechanism (presence of parasites in the migratory environment), so taking the left branch of the decision tree, considering a single mechanism, and then advocating for the specific mechanism (since the mechanism had not previously been included in models). This leads to an ‘Advocate’ (mechanism/novelty) persona, i.e. what are the consequences of including parasites in the second environment (which has previously been overlooked)? The results were fairly intuitive: having parasites in the second environment narrows the range of conditions where migration is favoured, and whether migration is favoured or not comes down to the details of risk of infection (how fast the different parasites are transmitted in each environment) and the cost of infection (how costly the two parasites are compared with each other). However, the authors also discovered that the model produced an intriguing result: migrants typically had lower infection prevalence while simultaneously having higher parasite diversity compared with non-migrants [33]. This result replicated an ongoing conflict in the empirical literature (described in the ‘Mediator’ section above’), and they decided to revise the paper framing accordingly. Revising the roadmap, this would now be a case of starting with pattern(s) (pattern 1 is that some empirical studies find that migrants have higher infection and pattern 2 is that other studies find the opposite), so taking the first right branch of the decision tree. At the next step, they were exploring known mechanisms underlying a set of patterns (since it is known empirically that parasites can be present in several environments), and finally at the next step, the focus was on reconciling conflicting patterns. This leads to a ‘Mediator’ persona (pattern/conflict). The authors felt that a pattern/conflict framing would allow their work to reach a broader audience than a mechanism/novelty one, so they wrote the paper around the question: ‘Here are conflicting empirical data patterns, how do we reconcile them?’.

## 4. Addressing misconceptions of theory

By providing a roadmap for how to formulate specific theoretical questions, we hope that our framework will pave the way for budding theorists to develop successful modelling projects. However, wading into theory can be daunting, particularly for empiricists, in no small part because there are many misconceptions about modelling that may discourage scientists from attempting their own theoretical work. Below, we use our framework to address these misconceptions by considering the example of R* theory—a seminal, mathematically derived hypothesis in community ecology that suggests that coexistence between resource competitors is possible when competitors are limited by different resources [55].

### (a) Misconception #1: simplifying assumptions prevent models from being useful

A misconception that appears in almost any context in which theory is discussed is the idea that models are not useful because they rely on simplifying assumptions. No model can include every phenomenon at work in a natural system, but we emphasize that this misconception misunderstands the purpose of theory. Theoretical models are meant to draw connections between informed assumptions and their outcomes. Models cannot exist without assumptions, so this criticism is non-sensical. Our framework focuses on the central modelling question rather than belabouring what a model omits. This type of reframing can help theory-wary scientists better understand the consequences of the assumptions they make. For example, R* theory is concerned with coexistence between species engaged in resource competition, and therefore, in its most basic formulation, ignores the effects of other types of competition like apparent competition [55]. While this type of interaction may be important in many natural systems, the predictions generated by R* remain useful for answering the central question regarding resource competition. Focusing on these assumptions as limitations of the model ignores the purpose of R*. If researchers, after considering the central theoretical question, find that their initial assumptions will prevent them from successfully addressing their question, we encourage them to amend or alter those assumptions—as some theorists have indeed done with R*, using it to consider the dynamics of apparent competition [56]. This could result in theoretical work that *advocates* for the importance of a change in assumptions.

### (b) Misconception #2: adding more model details always leads to predictive improvements

The freedom to amend modelling assumptions is key to successful theory, but that freedom often leads researchers to another misconception of theoretical work: the idea that a model can always be made more useful through the inclusion of additional details. It can be tempting to believe that the addition of another mechanism will make a model ‘truer to life’ and improve results. In practice, additional details increase mathematical complexity and make results harder to interpret. Expanding a model also comes with the cost of time and energy. Empiricists face a similar trade-off: while more factors can always be added to an experiment (or a broader range of observations collected), more data are not worth the effort spent if the central question is already answered by the data in hand. We suggest to new theoreticians that models should only be expanded in pursuit of the specific modelling question. Many such expansions would fall under the ‘Advocate’ persona, and thus should address why inclusion of novel mechanisms is important. When the aim of a model is prediction, it is more likely to be important to include additional phenomena [9,57]. For example, if one is interested in the robustness of R* coexistence when resources follow seasonal fluctuations, the inclusion of these dynamics would be justified [55]. Fluctuations in resource availability could also elucidate patterns of coexistence across temporal scales that R* is otherwise not situated to investigate [55]. Adding a system detail is worthwhile if it has the potential to: (i) change the predicted link between a model’s assumptions and its outcomes or (ii) open-up additional avenues of study through development of a new theoretical question. However, we emphasize that if the goal of a project is to increase understanding of a system, adding details beyond those sufficient to explain the pattern of interest will rarely improve theoretical insights [9,57]. For example, adding spatial structure and consumers of competitors to R* for the purpose of increasing biological realism could make the model intractable without improving understanding of conditions of coexistence between resource competitors [55]. We expect that our framework will help scientists new to theory make informed decisions about what to include and when to expand their models, though, in general, we recommend that models are kept as simple as possible.

### (c) Misconception #3: you can make a model show anything you want

Making decisions about what to include in a model can occasionally lead new practitioners of theory to believe that models can always be constructed to generate positive results supporting their preferred hypothesis. In some ways, this may be true: because theoretical models link assumptions and outcomes, if it is possible to think of a way that an assumption and an outcome can be connected, then a model can probably be constructed to reflect that. Generally, cases where this does not happen—i.e. when the process of model building uncovers a flaw in our logic—are not framed this way when published, which can contribute to the sense that models always end up supporting our initial hypotheses. However, a model that produces an intuitive result is often just a starting point for addressing precise theoretical questions. For example, the R* result that competitors can coexist on shared resources may seem trivial until one frames a more precise question, which could be done using any of the six framings. For example, using a pattern/robustness framing (the ‘Tinkerer’), one could ask ‘Under what conditions is coexistence between competitors possible?’ (rather than just ‘is coexistence possible’). Second, one could scale the understanding of this mechanism to an unlimited number of competing species (mechanism/novelty framing, the ‘Advocate’), and find that coexistence is theoretically possible as long as species are limited by different resources [55]. We suggest to new theoreticians that both getting precise on the question framing and the thoughtful addition of system details can increase the range of possible questions a model can answer, reducing how trivial a model feels. Our framework supports scientists in extending their initial assumptions into these types of precise, insightful questions.

### (d) Misconception #4: successful modelling requires extensive mathematical background

Finally, a misconception that represents a major barrier to the greater adoption of theoretical work in biology is the idea that extensive mathematical background is essential to developing and evaluating theoretical (especially mathematical) models. We believe that framing is much more important to successful theoretical work than mathematical acumen. This is because even practised theoreticians rarely have the need to create new models from scratch since the same mathematical representation can be translated into myriad biological meanings. Model development instead tends to consist of combining and altering pieces of existing models in interesting ways to answer new questions [17]. Since ‘most models are variants of previous models’ [17], there are many resources available to aid the mathematical evaluation of any biological model, such as Otto and Day’s ‘A Biologist’s Guide to Mathematical Models in Ecology and Evolution’ [58] and similar primers. Armed with these guides and the computational power of platforms like MATLAB or R, new practitioners of theory will be able to derive mathematically sound insights from models. However, because model development relies on altering existing models, we suggest that framing is essential to contextualize these models so that they can be appropriately amended for a researcher’s specific purpose. For example, amending R* theory to consider different functional responses in competing species would reflect the work of a ‘Tinkerer’, asking how robust the pattern of coexistence is to biologically relevant changes in the way the underlying mechanism is translated into mathematical terms. By understanding the framing of an existing model, researchers can appropriately adapt models to answer their own questions without the need for extensive mathematical training.

Theoretical modelling is an incredibly powerful research tool, one that is made richer and more useful by the inclusion of scientists who generally consider themselves empiricists. We hope that our framework, by addressing common misconceptions of theory, can assuage many of the anxieties that scientists new to theory may face and encourage them, particularly those early in their research careers, to take informed theoretical risks. As a final note, we emphasize that the utility of models does not hinge on their validation by empirical experiments or observation [12]. Models do not require data to be proven, in the same way that it is unnecessary to build a theoretical model to verify the results of an experiment done in the lab. Modelling is one way of knowing in science, most useful when paired with other types of investigation to improve our understanding of a given phenomenon. It is our hope that our framework helps scientists build generative questions to test with theory. Clarifying theoretical questions as we do here will make it easier to put modelling results in their proper context with findings from both laboratory and field work.

## 5. Conclusion

Here, we have argued that developing a clear verbal framing is critical when undertaking a new theoretical research project. Furthermore, we posit that the verbal framing is even more important than the mathematical framing when it comes to communicating the goal and results of a theoretical model with non-theorists, echoing a point from Ou *et al*. [14] on the importance of emphasizing the narrative reasoning behind models. Here, we present six types of verbal framings of theoretical questions that start with either a pattern or a mechanism, and that focus on novelty, robustness or conflict. Together, these lead to six personas that one might adopt as a theorist: the ‘Advocate’, ‘Explainer’, ‘Instigator’, ‘Mediator’, ‘Semantician’ and ‘Tinkerer’. We recognize that there are other ways to frame theory and hope this paper inspires discussion among those with other perspectives on how to frame theory in biology. We have shown that these framings also serve as tools to help avoid misconceptions of theoretical research. Finally, although our focus has been on the field of biology and the approach of mathematical theory, we expect that some of these ways of thinking in terms of being explicit about pattern and mechanism apply broadly to other fields as well (e.g. [59]). In sum, we hope the six personas we present here can serve as blueprints that researchers who are relatively new to theoretical research can use when developing their theoretical research questions.

## Data Availability

This article has no additional data.
